# Validation of the Spanish Version of the ICECAP-O for Nursing Home Residents with Dementia

**DOI:** 10.1371/journal.pone.0169354

**Published:** 2017-01-09

**Authors:** Carmen M. Sarabia-Cobo, Paula Parás-Bravo, Francisco José Amo-Setién, Ana Rosa Alconero-Camarero, María Sáenz-Jalón, Blanca Torres-Manrique, Raquel Sarabia-Lavín, Angela Fernández-Rodríguez, Tamara Silio-García, Rosario Fernández-Peña, María Paz-Zulueta, Miguel Santibáñez-Margüello

**Affiliations:** Department of Nursing, University of Cantabria, Cantabria, Spain; University of Glasgow, UNITED KINGDOM

## Abstract

**Background:**

Measurement of health-related quality of life (HRQoL) is important for a chronic disease, such as dementia, which impairs the quality of life of affected patients in addition to their length of life. This is important in the context of economic evaluations when interventions do not (only) affect HRQoL and these other factors also affect overall quality of life.

**Objective:**

To validate the Spanish translation of the ICECAP-O’s capability to measure Health-related quality of life in elderly with dementia who live in nursing homes.

**Method:**

Cross-sectional study. For 217 residents living in 8 Spanish nursing homes, questionnaires were completed by nursing professionals serving as proxy respondents. We analyzed the internal consistency and other psychometric properties. We investigated the convergent validity of the ICECAP-O with other HRQoL instruments, the EQ-5D extended with a cognitive dimension (EQ-5D+C), the Alzheimer’s Disease Related Quality of Life (ADRQL) measures, and the Barthel Index measure of activities of daily living (ADL).

**Results:**

The ICECAP-O presents satisfactory internal consistency (alpha 0.820). The factorial analysis indicated a structure of five principal dimensions that explain 66.57% of the total variance. Convergent validity between the ICECAP-O, EQ-5D+C, ADRQL, and Barthel Index scores was moderate to good (with correlations of 0.62, 0.61, and 0.68, respectively), but differed between dimensions of the instruments. Discriminant validity was confirmed by finding differences in ICECAP-O scores between subgroups based on ADL scores (0.70 low, 0.59 medium, and 0.39 high level care), dementia severity (0.72 mild, 0.63 medium, and 0.50 severe), and ages (0.59 below 75 years and 0.84 above 75 years).

**Conclusions:**

This study presented the first use of a Spanish version of the ICECAP-O. The results indicate that the ICECAP-O appears to be a reliable Health-related quality of life measurement instrument showing good convergent and discriminant validity for people with dementia.

## Introduction

Population aging is a phenomenon of social importance caused by reduced mortality, birth control, and increased life expectancy [1]. In Spain, this aging process has occurred more rapidly than global averages; the population over 65 years old increased from 14.95% in 1995 to 18.4% in 2016, and life expectancy, at 83.3 years, is among the highest in the European Union [2].

This has given way to increased degenerative diseases, including dementia. The changes that occur in dementia include a loss of function that makes the affected more dependent on others for the performance of activities [3] [4]. These types of diseases alter the quality of life of affected individuals due to the changes that occur at the physical, social, psychological, and even spiritual levels [3]. In the latest study in Spain the estimated prevalence of dementia in individuals aged 65 and older in Madrid, was 61.7% (confidence interval [CI] 95%, from 58.4 to 65.1). Alzheimer's disease was presented with a prevalence of 16.9% (95% CI, 14.3 to 19.5) [5].

Frequently, in advanced stages of dementia, a sufficient amount and quality of professional care can only be provided in an institutional long-term care setting, making admissions inevitable for a growing number of elderly with dementia [6]. Two-thirds of older people living in nursing homes in Spain have dementia [7]. Faced with increasing demand, the long-term care sector in many countries may experience strong budgetary pressures and increased questions about optimal resource allocation and affordability of care [8].

Dementia has substantive effects on healthy aging as it can negatively impact one’s health-related quality of life (HRQoL) [9]. Health-related quality of life has been defined as an individual’s subjective experience of the impact that illnesses and their treatments have on the individual’s functioning in a variety of domains, such as physical, psychological and social functioning, as well as the impact of illnesses on the ability to engage in daily activities [9]. Broader HrQoL measures, often named wellbeing measures, should be used to capture more facets of peoples’ lives than health status alone. In the context of providing long-term care for those with dementia, HRQoL is important outcomes to assess and monitor [10]. In fact, HRQoL is identified as an important outcome in interventions aimed at combatting cognitive dysfunction [11]. In dementia, determining HRQoL is limited by cognitive disorders and incorporates external elements in relation to the activities and remaining positive behaviors [12]. Evaluating HRQoL in dementia is feasible in other disorders with similar effects, although in advanced stages of dementia the patient self-assessment is replaced by a reliable informant. HRQoL is a measure used for various reasons, such as to measure the impact of care and treatment received, the quality of care, the evolution of the disease itself, and the economic impact [13] [14] [15]. In economic evaluations, benefits are frequently assessed by changes in HRQoL combined with the duration an individual spends in various health states. Economic evaluation is increasingly used in in the health sector as a decision support tool for resource allocation, but may aid the allocation of resources in long-term care as well [16] [17].

HRQoL is most commonly measured with the EQ-5D instrument [18]; however, Euro Quality of Life 5D (EQ-5D) is a very generic measurement instrument that only takes HRQoL into account and is therefore not sufficient for a complete economic evaluation in the case of patients with dementia [17]. Other more specifics instruments, such as Alzheimer Disease-related Quality of Life (ADRQL) [19] [20], include further aspects of a patient’s life but do not take into account physical health and do not allow for comparison across different diseases [17] [21]. This is of particular relevance to the nursing home population, where older people typically suffer from a range of co-morbidities [21], making it difficult to perform a complete assessment of the impact of specific interventions using disease-specific HRQoL instruments alone.

The most widely applied older-person specific instrument to measure quality of life is the ICECAP-O (Investigating Choice Experiments for the Preferences of Older People) [22, 23], a relatively new developmental instrument [24]. ICECAP-O seems to satisfy the requirements of economic evaluation in the case of dementia [22] [23]. It is intended as an outcome that measures economic evaluations of both health and social services, where beyond health, wellbeing aspects also have to be considered. ICECAP-O is an instrument with a broader perspective than HRQoL and is conceptually based on the capability approach [25]. It was originally developed in the UK for use in the economic evaluation of health and social care interventions [22] and to cover the domains of attachment (love and friendship), security (thinking about the future without concern), role (doing things that make you valued), enjoyment (pleasure), and control (independence). Validation studies confirmed that the ICECAP-O evaluates a spectrum of outcomes beyond HRQoL [26]. Until now, the ICECAP-O was used in the general population in the UK [22] and Australia [27], in a psychogeriatric nursing home setting in the Netherlands [28], in Dutch psycho-geriatric nursing homes [29], in nursing home residents with dementia in German [30], in a population of post-hospitalized older adults in the Netherlands [31], and among older adults with mobility impairments in Canada [32]. After carrying out a systematic review and contacting the original authors of the instrument [22, 23], to date, the ICECAP-O has not been validated in a population of patients with dementia in nursing homes in Spain.

The aim of this study to validate the Spanish translation of the ICECAP-O’s capability to measure Health-related quality of life in elderly with dementia who live in nursing homes.

## Methods

### Design, Setting, Study Population, and Data Collection

A cross-sectional study was conducted in 8 nursing homes belonging to the same foundation spread over 6 cities in Spain. They involved 217 people with dementia in moderate stages who were older than 65 and had been living in the nursing home for longer than 3 months. Fourteen nursing professionals of each nursing home were asked to complete the questionnaire in the manner that the patient would have if he/she were able to answer the questions. In all the cases, five researchers collected all the data, interviewing all patients and the collaborating nurses when needed.

Inclusion criteria: over 65 years of age and institutionalized for more than 3 months, presenting a diagnosis of dementia with over six months of evolution, classified according to the GDS (inclusion levels 4 to 6 of the Global Deterioration Scale (GDS) [33]. This classification should be made by the neurologist who diagnosed or followed the evolution of the patients.

The study was approved by the Bioethics Committee of the Foundation in Madrid (COD. 2016.08) and at all directions of the centers. Informed consent was obtained from legal guardians for all participants after they were informed verbally and in writing. The principal investigator is responsible for keeping informed consent.

Sample size. The sample size was estimated with a programme for calculating sample size and the power of a contrast of hypothesis (Granmo v. 7.11, developed by Institut Municipal d'Investigació Mèdica, Barcelona, Spain), for a significance level of 0.05 and a power of 0.8, assuming equal variances). Adjusting for a dropout rate of 20%, it was determined that 208 people were needed.

### Instruments

Different socio-demographic variables of patients were collected, such as age, sex, marital status, and length of stay in the nursing home.

**The Global Deterioration Scale (GDS)** [33] assesses the degree of dementia. This scale enables the evolution of cognitive impairment from normal aging to a person with dementia. It consists of seven phases, ranging from "zero cognitive impairment" to "very severe dementia." The first two levels (GDS 1 and 2) correspond to normal states. The GDS 3 is the first phase in which there are clear deficits, although it is not considered sufficient to diagnose dementia. GDS 4, GDS 5, GDS 6, and GDS 7 reflect a cognitive impairment linked to dementia (mild, moderate, and severe dementia, respectively).

**The ICECAP-O** [22] [23] measures capability wellbeing using five domains or attributes (attachment, security, role, enjoyment, and control), with one question per dimension. Distinguishing four levels within each domain (levels generally range from all, a lot, a little, to not any; exact wording of levels varies per dimension). Each level distinguishes 1,024 possible “capability states.” The ICECAP-O tariffs have values between 0 (no capabilities) and 1 (full capabilities). The ICECAP-O is a five-item multiple choice questionnaire where each attribute has 4 possible response options. For this first use of the ICECAP-O in Spanish, the instruments' original author's/recommended translation guidelines were fully met. The original ICECAP-O version, as developed by Coast et al., [23] was forward-backward translated from English into Spanish by two independent translators. Subsequently it was translated back into English by two independent translators. The last version in English was sent to the original authors of the application for its validation.

In order to compute capability values, the British tariffs were applied because these do not exist for Spain. Lower scores thus represented fewer preference-based capabilities. The complete explanation as to how to calculate them was fully described on the ICECAP website: http://www.icecap.bham.ac.uk/tariffs.shtml.

**Alzheimer Disease Related Quality of Life (ADRQL)** [19]. We used the revised 40-item version that allows for the assessment of QoL for people with mild, intermediate, or late-stage dementia using proxy responses [34]. The dementia-specific, multi-dimensional ADRQL instrument can be completed by patients’ professional caregivers [20]. The ADRQL measures the dimensions of social interaction, awareness of self, enjoyment of activities, feelings and mood, and response to surrounding. The various dimensions range from 4 to 12 items on a dichotomous scale and each item is weighted in a range between 9.15 and 13.75, based on a judgment of importance by caregivers [20]. For each dimension, a separate subscale can be calculated and summed up in one total score ranging from 0 (lowest quality of life) to 100 (highest quality of life) [19]. The instrument exhibits good psychometric properties with adequate validity, good internal-consistency reliability, very low missing data, and good sensitivity to change [20] [34].

**The EQ5D** [18] Spanish version [35] measures HRQoL in terms of five dimensions (mobility, self-care, daily activities, pain and discomfort, and anxiety and depression) with three levels each (1 = no problems, 2 = moderate problems, and 3 = extreme problems) describing 243 health states. For use in people with dementia, the EQ-5D was extended with a cognitive dimension (EQ-5D+C), for which utility scores are unavailable [35] [36]. The health index component of the EQ-5D+C is therefore made up of six dimensions: mobility, self-care, usual activities, pain/discomfort, anxiety/depression, and cognition. Each dimension has three levels: no problems, some problems, and extreme problems.

**The Barthel-Index** [37], Spanish version [38] has 10 items of activities of daily living (ADL): feeding, grooming, bathing, dressing, bowel and bladder care, toilet use, ambulation, transfers, and stair climbing. The total score ranges from 0 to 10. The total score thus ranges between 0 (completely dependent) and 100 (completely independent) with a cutoff score of 65 indicating need for ADL assistance, with a classification in low (40–65), medium (20–40), and high (<20) care dependency groups. This is the most common test that measures residents’ abilities to perform activities of daily living by proxy- or self-report.

### Hypotheses

To establish convergent validity we expected moderate to strong and positive correlations between the ICECAP-O, the EQ-5D+C, and ADRQL (these instruments measure partial operationalizations of QoL) (H1). Furthermore, we expected a moderate and positive correlation between the ICECAP-O dimension and tariff scores and the Barthel Index (ADL) (H2). For discriminant validity, we expected to find differences in ICECAP-O tariff scores between residents suffering from severe, moderate, and mild/moderate dementia (based on the GDS), between ADL dependent (Barthel score <65) and ADL independent (Barthel score >65) residents between low, medium, and high care dependency groups (H3). A higher score on the ICECAP-O was expected for the better-off groups.

### Data Analysis

The construct validity of the ICECAP-O questionnaire was calculated through factor analysis using principal component extraction and Varimax rotation with Kaiser. Each variable was included in a single factor, according to their factor loading, by setting minimum saturation criteria of 0.50. Varimax rotation is assumed to be the most appropriate, since it is expected to discriminate between the maximum number of factors forming the scale. Estimates of sampling adequacy for Kaiser-Meyer-Olkin (KMO; range 0–1) and Barlett statistical significance (if its value is close to the unit and they are significant with p <0.05, indicating that the analysis with reduced variables is appropriate) were calculated. The reliability of the questionnaire was calculated by analyzing the internal consistency, using Cronbach Alpha, which should be interpreted as an indicator of the internal consistency of the items, because it is calculated from the covariance between them.

The demographic characteristics of residents and proxies were analyzed using descriptive statistics. Mean or median, standard deviation, were computed from the demographic data of the residents and for the different dimensions of the instruments. Correlations between the outcomes of the ICECAP-O and dimensions of the ADRQL, EQ-5D+C, and ADL were used to estimate convergent validity. Discriminant validity was analyzed using a t-test and one-way ANOVA to explore differences in means of the ICECAP-O between different demographic and dementia-related groups. For all analyses the level of significance was set at p<0.05. Data was analyzed using SPSS v.22.0.

## Results

### Reliability Analysis and Questionnaire Validity

Estimates of sampling adequacy of Kaiser-Meyer-Olkin (KMO = 0.877) and Bartlett statistical significance (p = 0.000) were calculated. Communalities analysis indicated that all items are above 0.5, and then all questions were accepted as valid to form part of the questionnaire. Using this method, the principal components’ 5 major components or the factors with eigenvalues greater than 1 were obtained, which account for 66.57% of the total variance, and an explanation percentage at appropriate acceptance levels was obtained (see Table 1).

**Table 1:** Total variance explained.

Component	Rotation sums of squared loadings		
	Total	% of variance	% cumulative
**1**	5.48	21.21	21.21
**2**	2.26	14.78	36.00
**3**	2.10	12.47	48.47
**4**	1.91	9.54	58.02
**5**	1.84	8.54	66.57

These factors were classified as the same 5 domains or attributes as the original version [21]: attachment, security, role, enjoyment, and control. One item was included in a single factor, according to their factor loading, by setting saturation minimum criteria at 0.30. Rotated factor solutions according to VARIMAX formed a well-defined structure without overlapping. To assess the internal consistency of the questionnaire a Cronbach alpha value of 0.820 was obtained. This value was not improved by eliminating some of the items, therefore all items were maintained. The values of item-total correlation ranged between 0.52 and 0.73.

### Demographics and Descriptive Characteristics

Data were collected between March and June 2016. The questionnaires were collected in 8 nursing homes by 14 nurses from a total of 217 patients with mid-stage dementia. Table 2 shows the sociodemographic variables, the characteristics of the residents, the measurement instruments, and their dimensions.

**Table 2:** Demographic and description measurement instruments (N = 217).

Variables	% Mean/SD
Age	87.05 (5.8)
Sex	80.6%female
**GDS**	
• Mild (4)	10.2%
• Moderate (5)	32.6%
• Severe (6/7)	57.2%
**Months living in nursing home**	
• 3 ≤ 6 months	3.7%
• 6 ≤ 12 months	7.5%
• 12 ≤ 24 months	%
• > 24 months	69.4%
**Marital Status**	
• unmarried	4.7%
• married	18.7%
• divorced	7.2%
• widowed	69.4%
**ICECAP-O**	
• Attachment	(0.62) 3
• Security	(0.94) 3
• Role	(0.88) 2
• Enjoyment	(0.63) 3
• Control	(0.93) 1
• ICECAP Tariffs	0.61 (0.77)
**EQ-5D+C**	
• Mobility	(0.92) 2
• Self-Care	(0.64) 3
• Usual activities	(0.82) 3
• Pain/Discomfort	(0.92) 1
• Anxiety/Depression	(0.88) 1
• Cognition	(0.64) 3
• EQ-5D+C Tariffs	0.66 (0.57)
**ADRQL (Original weights)**	
• Social Interaction (SI)	(22.69)
• Awareness of Self (AS)	(17.36)
• Feelings and Mood (FM)	(21.87)
• Enjoyment of Activities (EA)	(31.58)
• Response to Surroundings (RS)	(21.36)
• Overall ADRQL	73.39 (22.97)
**Barthel-Index (ADL)**. **Care level**	
• Overall ADL	(28.56)
• Low	17.9%
• Medium	30.8%
• High	51.3%

The overall average scores for the instruments: average ICECAP-O score (based on the tariffs) was 0.61, EQ-5D score was 0.66, and the ADRQL score (based on tariffs) was 73.39.

### Convergent Validity

Table 3 shows that the ICECAP-O scores were strongly correlated with EQ-5DC scores, ADRQL scores, and Barthel scores. Correlations between the ICECAP-O tariff scores and the different dimensions of the EQ-5D+C were strong and significant. Correlations between the ICECAP-O and the ADRQL proved to be similarly strong and significant.

**Table 3:** Convergent Validity.

	ICECAP -O tariff	ICECAP-O dimension scores				
		Attachment	Security	Role	Enjoyment	Control
**Barthel-Index (ADL)** Score	0.68**	0.25*	0.34*	0.72**	0.43**	0.86**
**EQ-5D+C** Utilities	0.62**	0.11*	0.32*	0.71**	0.56**	0.41**
**EQ-5D+C** Dimension Scores						
• Mobility	0.59**	0.21	0.002	0.68*	0.47**	0.32*
• Self-Care	0.66*	0.34**	-0.03	0.69*	0.37**	0.78**
• Usual Active	0.45**	0.69*	0.06	0.41**	0.27**	0.66**
• Pain/Discomfort	0.36*	0.21	-0.002	0.35**	0.42	0.65*
• Anxiety/ Depress	0.002	0.36**	0.14*	0.54	0.47**	0.004
• Cognition	0.54**	0.02	-0.05	0.36**	0.47**	0.35**
**ADRQL** Overall	0.61**	0.27*	0.23	0.45**	0.36	0.14**
**ADRQL** Dimension Scores						
• Social Interaction (SI)	0.54**	0.65**	0.21	0.37	0.47*	.046**
• Awareness of Self (AS)	0.02**	0.47**	-0.03	0.36**	0.47*	0.25**
• Feelings and Mood (FM)	0.35*	0.54**	0.57**	0.28**	0.09	0.36*
• Enjoyment of Activities (EA)	0.23**	0.65*	-0.21	0.03	0.38**	0.43**
• Response to Surroundings (RS)	0.21*	0.36*	0.55*	0.47**	0.26*	0.34**

*Significance on the 5% level;

**Significance on the 1% level.

The individual ICECAP-O dimensions of role and control were strongly and significantly correlated with the EQ-5D+C and to a lesser extent with dimension mobility and self-care, usual activities, pain/discomfort, and cognition. Attachment was significantly and strongly correlated with SI, FM, and EA (ADRQL). The Barthel index was significantly correlated with all ICECAP-O dimensions with correlations between the Barthel index and the role and control dimensions being particularly strong.

### Discriminant Validity

In Table 4, the results of the ANOVA showed significant differences in ICECAP-O scores between patients with different dementia severity and ages (i.e., above or below 75). ANOVA results showed that the ICECAP-O tariff scores differentiated between residents classified into different care dependency levels. As expected, lower scores were observed for the more severe groups, and higher scores for the less severe groups.

**Table 4:** Discriminant Validity.

**Severity**	**mildmean**	**moderate**	**severe**	**p-value**
	mean (SD)	mean (SD)	mean (SD)	
**EQ-5D+C**	0.71 (0.14)	0.62 (0.31)	0.31 (0.17)	0.000**
**ICECAP-O**	0.72 (0.11)	0.63 (0.21)	0.50 (0.10)	0.000**
**ADRQL**	77.02 (13.58)	73.21 (11.22)	62.74 (17.50)	0.000**
**Age**	below 75		above 75	p-value
	mean (SD)		mean (SD)	
**EQ-5D+C**	0.31 (0.17)		0.90 (0.21)	0.005**
**ICECAP-O**	0.59 (0.22)		0.84 (0.09)	0.000**
**ADRQL**	68.25 (17.52)		80.02 (16.21)	0.000**
**Care level (ADL)**	low	medium	high	p-value
	mean (SD)	mean (SD)	mean (SD)	
**EQ-5D+C**	0.74 (0.11)	0.60 (0.11)	0.32 (0.18)	0.000**
**ICECAP-O**	0.70 (0.19)	0.59 (0.14)	0.39 (0.12)	0.000**
**ADRQL**	74.11 (15.18)	71.45 (18.23)	59.77 (14.25)	0.000**

**Significance on the 1% level

## Discussion

In this study, the ICECAP-O was used and validated for the first time in Spain in specialized nursing homes for people with dementia. Our results indicated that the ICECAP-O has good psychometric properties and convergent and discriminant validity.

Hypotheses were supported by the significant and strong correlations of the ICECAP-O tariff scores with HRQoL scores (both EQ-5D+C and ADRQL scores) (H1) and with ADL scores (H2). Moreover, as hypothesized (H3), the ICECAP-O significantly discriminated between subgroups based on dementia severity (mild, moderate, and severe), ADL status (care levels of low/middle/high) and age (residents <75 and >75 years), thus supporting discriminant validity. The results provide support that the ICECAP-O measures dimensions relevant for people with dementia and therefore a broader spectrum than only health, as measured by the EQ-5D.

### Methodological Limitations

This study has a number of limitations worth mentioning. First, the residents for this study were not randomly selected and therefore might have characteristics that differ from the typical population with dementia in Spain nursing homes. However, this restriction may only influence the results to a certain extent, because the focus of the study was the validation of the properties of the quality of life measurement instruments and the current sample seems adequate. Second, British tariffs were used for the ICECAP-O since Spanish tariffs are not yet available. For this reason, results may be imprecise because weights for capability dimensions might vary between countries.

### Convergent Validity and Discriminant Validity

The average ICECAP-O tariff score found in this study within a nursing home setting (0.61) was comparable to the scores reported in a Dutch study performed in nursing homes (0.63) [28] and in a German study also performed in nursing homes (0.63) [30], and was substantially lower than the score for community-living elderly, where the average scores ranged between 0.81–0.84 [22] [27] [31] [32] [37–41]. These findings support the reliability of our results.

The finding of the strong correlation between the ICECAP-O dimensions and the EQ-5D dimensions shows that physical health is captured to a wide extent by the ICECAP-O capability measurement, which is consistent with other findings [18] [28–32]. Overall, we found significant correlations between the ICECAP-O dimensions and the individual EQ5D+C dimensions, with the exception of attachment, which was not significantly correlated with the mobility, pain/discomfort, and cognition dimensions of the EQ5D+C. Security was only significantly correlated with the EQ5D dimension of anxiety/depression. This can be explained because people with dementia, because the disease, lose their sense of security [41] [42].

Role and control correlated significantly with all EQ5D+C dimensions with the exception of Anxiety/Depression, which could be because people with dementia, especially in moderate and advanced stages, have anxiety disorders or depression treated with non-pharmacological and pharmacological measures [43]. These findings are in line with other similar studies [28–31]. The results confirmed the expected significant correlation between the ICECAP-O and the ADRQL scores, which shows that the ICECAP-O captures both generic HRQoL and dementia specific quality of life. The correlation between ADL and ICECAP-O scores reflected that a loss of independence in ADL was associated with a decline in wellbeing. This correlation is strengthened by the same finding for the other applied quality of life measurement instruments in this study, confirming previous results that reduced ADL leads to a decrease in quality of life [44]. These significant findings point in the direction of favorable convergent validity.

The ICECAP-O dimension “security” is an exception to the other dimensions because it showed almost no significant correlation with the majority of dimensions of the other quality of life measures. The same finding was observed in the Dutch and German ICECAP-O validation studies in which no correlation with the dimension “security” was found [29, 30]. This might be because people with dementia do not generally worry about the future past a certain point, [41]. On the other hand, the moderately to highly significant correlations of the capability dimensions “enjoyment,” “role,” and “control” with the ADRQL and the EQ-5D+C dimensions support the hypothesis that the ICECAP-O captures dimensions that are relevant for the elderly. Such correlations with the EQ-5D were also found in the general British population [22] and in the German nursing homes [30] [41].

The ICECAP-O discriminated between patients based on the variables age, dementia severity, and ADL. Therefore, the group of healthier residents and the group with a higher functional status also reached the expected higher tariff scores. This suggests that the ICECAP-O is sensitive to age differences and to indicators of health, confirming that reasonable discriminant validity exists.

The ICECAP-O is a measure of wellbeing, and therefore has the potential to broaden the evaluative space of economic evaluations in health care by focusing on more than health alone. This is a particularly useful property in case populations such as institutionalized patients with dementia, distinguished by decreasing independence and multi-morbidity, potentially across different health dimensions.

Further research is needed to confirm the current favorable findings and to further explore the feasibility, validity, and usefulness of the ICECAP-O instrument, as well as in the context of economic evaluations. More research is especially encouraged in more homogeneous populations characterized by a single disease, since dementia is a syndrome with very heterogeneous diseases (eg. Alzheimer, Lewy bodies, etc).

## Conclusion

This study presented the first use of a Spanish version of the ICECAP-O. The results indicate that the ICECAP-O appears to be a reliable wellbeing measurement instrument showing good convergent and discriminant validity for people with dementia. The psychometric properties are adequate, and we confirmed 5 dimensions and a good internal consistency of the questionnaire (Cronbach alpha = 0.820).

Future research is required to confirm these findings in other settings and similar samples, with particular attention paid to dementia severity, diagnosis, and validation alongside dementia specific quality of life measures.

## Supporting Information

S1 Table The ICECAP-O instrument proxy version. (DOCX)

S2 Table Spanish version of the ICECAP-O. (DOCX)

## Abbreviations

ADL: Barthel Index of activities of daily living
ADRQL: Alzheimer Disease Related Quality of Life
EQ-5D: Euroqol
GDS: Global deterioration Scale
HRQoL: Health-related quality of life
ICECAP-O: ICEpop CAPability measure for older people
Qol: Quality of life
