# Supplementary material for: Validation of the Spanish Version of the ICECAP-O for Nursing Home Residents with Dementia
Source: PLoS One. 2017 Jan 9;12(1):e0169354. doi: 10.1371/journal.pone.0169354 (PMC5222189; doi:10.1371/journal.pone.0169354)
Supplement: S1 Table — (DOCX) [file pone.0169354.s001.docx]

**Appendix S1** The ICECAP-O instrument proxy version.

By placing a tick (V) in ONE item in each SECTION, indicating what which statement best describes your quality of life at the moment.

1. Love and Friendship

| I can have all of the love and friendship I want | 4 |
| --- | --- |
| I can have much love and friendship I want | 3 |
| I can have a bit of love and friendship I want | 2 |
| I cannot have any of the love and friendship I want | 1 |

2. Thinking about the future

| I can think about the future without any concern | 4 |
| --- | --- |
| I can think about the future with some concern | 3 |
| I can only think about the future with some concern | 2 |
| I can only think about the future with a lot of concern | 1 |

3. Doing things that make you feel valued

| I am able to do all the things that make me feel valued | 4 |
| --- | --- |
| I am able to do many of the things that make me feel valued | 3 |
| I am able to do some of the things that make me feel valued | 2 |
| I am unable to do any of the things that make me feel valued | 1 |

4. Enjoyment and pleasure

| I can have all the enjoyment and pleasure that I want | 4 |
| --- | --- |
| I can have a great deal of the enjoyment and pleasure that I want | 3 |
| I can have a bit of the enjoyment and pleasure that I want | 2 |
| I cannot have the enjoyment and pleasure that I want | 1 |

5. Independence (Unassisted)

| I am able to be completely independent | 4 |
| --- | --- |
| I am able to be independent in many things | 3 |
| I am able to be independent in a few things | 2 |
| I am unable to be completely independent | 1 |
