# Supplementary material for: Validation of the Spanish Version of the ICECAP-O for Nursing Home Residents with Dementia
Source: PLoS One. 2017 Jan 9;12(1):e0169354. doi: 10.1371/journal.pone.0169354 (PMC5222189; doi:10.1371/journal.pone.0169354)
Supplement: S2 Table — (DOCX) [file pone.0169354.s002.docx]

**Appendix S2** Spanish version of the ICECAP-O.

SOBRE SU CALIDAD DE VIDA

Marque con una (V) UNA casilla en cada APARTADO, indicando qué frase expresa mejor su calidad de vida en estos momentos.

1. Amor y amistad

| Puedo tener todo el amor y la amistad que yo quiero | 4 |
| --- | --- |
| Puedo tener gran parte del amor y la amistad que yo quiero | 3 |
| Puedo tener un poco del amor y la amistad que yo quiero | 2 |
| No puedo tener el amor y la amistad que yo quiero | 1 |

2. Pensando en el futuro

| Puedo pensar en el futuro sin ninguna preocupación | 4 |
| --- | --- |
| Puedo pensar en el futuro con un poco de preocupación | 3 |
| Solo puedo pensar en el futuro con cierta preocupación | 2 |
| Solo puedo pensar en el futuro con mucha preocupación | 1 |

3. Hacer las cosas que te hacen sentir valorado

| Soy capaz de hacer todas las cosas que hacen que me sienta valorado/a | 4 |
| --- | --- |
| Puedo hacer muchas de las cosas que hacen que me sienta valorado/a | 3 |
| Puedo hacer alguna de las cosas que hacen que me sienta valorado/a | 2 |
| No puedo hacer ninguna de las cosas que hacen que me sienta valorado/a | 1 |

4. Disfrute y placer

| Puedo tener todos el disfrute y el placer que yo quiero | 4 |
| --- | --- |
| Puedo tener una gran parte del disfrute y el placer que yo quiero | 3 |
| Puedo tener un poco del disfrute y el placer que yo quiero | 2 |
| No puedo tener el disfrute y el placer que yo quiero | 1 |

5. Independencia (SIN necesidad de ayuda)

| Soy capaz de ser completamente independiente | 4 |
| --- | --- |
| Soy capaz de ser independiente en muchas cosas | 3 |
| Soy capaz de ser independiente en algunas cosas | 2 |
| Soy incapaz de ser completamente independiente | 1 |
